# *In vivo* MRI Successfully Reveals the Malformation of Cortical Development in Infant Rats

**DOI:** 10.3389/fnins.2020.00510

**Published:** 2020-05-20

**Authors:** Minyoung Lee, Eun-Jin Kim, Dong-Cheol Woo, Woo-Hyun Shim, Mi-Sun Yum

**Affiliations:** ^1^Department of Pediatrics, Asan Medical Center, Asan Medical Institute of Convergence Science and Technology, University of Ulsan College of Medicine, Seoul, South Korea; ^2^Asan Institute for Life Sciences, Asan Medical Center, Seoul, South Korea; ^3^Department of Radiology, Asan Medical Center Children’s Hospital, University of Ulsan College of Medicine, Ulsan, South Korea

**Keywords:** malformations of cortical development (MCD), methylazoxymethanol (MAM), animal model, GABAergic neurons, GluCEST, *in vivo* MRI, infant rats

## Abstract

**Objective:** Malformations of cortical development (MCDs) are major causes of intractable epilepsies. To characterize the early neuroimaging findings of MCDs, we tried to identify the MRI features consistent with pathological findings in an infant rat MCD model, prenatally exposed to methylazoxymethanol (MAM), by using newly developed MRI techniques.

**Methods:** At gestational day 15, two doses of MAM (15 mg/kg intraperitoneally) or normal saline were injected into pregnant rats. The offspring underwent *in vivo* MRI, including glutamate chemical exchange saturation transfer (GluCEST), ^1^H-MR spectroscopy, and diffusion tensor imaging, at postnatal day (P) 15 using a 7T small-animal imaging system. Another set of prenatally MAM-exposed rats were sacrificed for histological staining.

**Results:** At P15, the retrosplenial cortex (RSC) of rats with MCDs showed decreased neuronal nuclei, parvalbumin, and reelin expressions. Moreover, dendritic arborization of pyramidal cells in the RSC significantly decreased in infant rats with MCDs. *In vivo* MRI showed significantly decreased GluCEST (%) in the RSC of rats with MCDs (*p* = 0.000) and a significant correlation between GluCEST (%) and RSC thickness (*r* = 0.685, *p* = 0.003). The rats with MCDs showed reduced glutamate (*p* = 0.002), *N*-acetylaspartate (*p* = 0.002), and macromolecule and lipid levels (*p* = 0.027) and significantly reduced fractional anisotropy values in the RSC.

**Conclusion:** *In vivo* MRI revealed reduced neuronal population and dendritic arborization in the RSC of infant rats with MCDs during the early postnatal period. These pathological changes of the cortex could serve as clinical imaging biomarkers of MCDs in infants.

## Introduction

The cerebral cortex is composed of six layers of glutamatergic and inhibitory interneurons (Kwan et al., 2012). The migration of these neurons into the proper layer of the cerebral cortex is an essential process during early cortical development, and its disruption causes malformations of cortical development (MCDs). MCDs are a broad spectrum of diseases caused by genetic or environmental insults (Colciaghi et al., 2011; Guerrini and Dobyns, 2014) and are associated with many neurological diseases, including developmental delay and intractable epilepsies (Kelsom and Lu, 2013; Wamsley and Fishell, 2017).

In particular, MCDs are the most common cause of pediatric intractable epilepsy (Barkovich et al., 2015; Iffland and Crino, 2017; Kim et al., 2017), and epilepsy surgery is the only curative treatment option because of the poor response to anticonvulsant drugs (Colciaghi et al., 2011; Barkovich et al., 2015). However, in clinical settings, localization of MCDs for epilepsy surgery is not always possible with current imaging techniques, especially in infants or in individuals with small focal cortical dysplasia (FCD). In addition, many patients with FCD type I are diagnosed only after the surgical excision of epileptic foci, and some of them experience surgical failures due to incomplete resection (Choi et al., 2018; Chen et al., 2019). Thus, non-invasive imaging diagnosis of FCD is important to offer the right therapeutic option to patients with intractable focal epilepsies (Jayalakshmi et al., 2019).

Various animal models of MCDs have been used for translational research (Kuzniecky, 2015; Luhmann, 2016), and the methylazoxymethanol (MAM) model is one of them. The offspring of MAM-treated rats are affected by developmental brain abnormalities similar to those observed in patients with MCDs (Chevassus-Au-Louis et al., 1999; Colacitti et al., 1999; Luhmann, 2016; Kim et al., 2017). Previously, our group reported *in vivo* anatomical disruption as well as increased spasm susceptibility, cognitive impairment, and abnormal cortical electrical activities in this MAM-induced MCD rat model (Kim et al., 2017).

Using this MAM-induced MCD rat model, we first tried to analyze the pathological characteristics of MCDs during infancy, and then to determine whether the MCD cortex can be distinguished from normal tissue by using newly developed brain MRI techniques.

## Materials and Methods

### Animals

**FIGURE 1:**
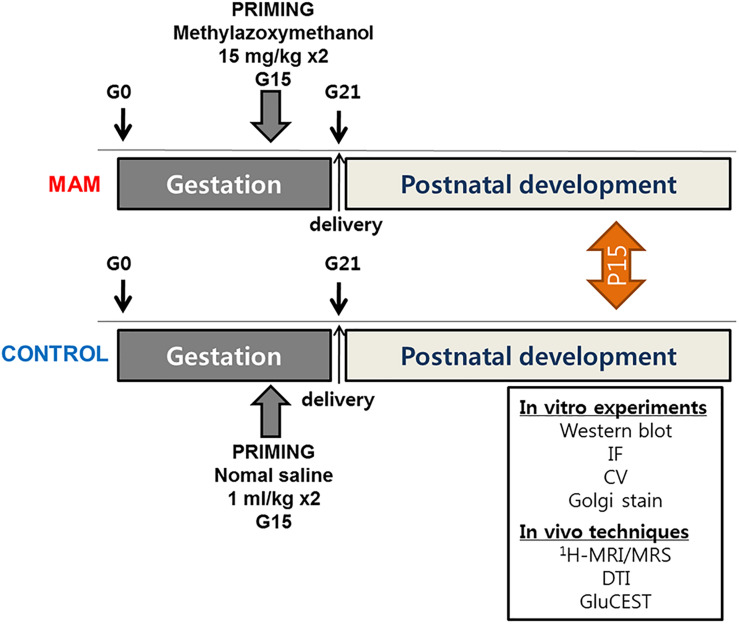
The timeline of experimental procedures. MRS, MR spectroscopy; DTI, diffusion tensor imaging; GluCEST, glutamate chemical exchange saturation transfer; MAM, methylazoxymethanol; IF, immunofluorescence staining; CV, cresyl violet staining.

The experiments were approved by the Institutional Animal Care and Use Committee of the Ulsan University College of Medicine and conformed to the Revised Guide for the Care and Use of Laboratory Animals (8th Edition, 2011). Timed-pregnant Sprague-Dawley rats were purchased (Orient Bio Inc., Seoul, Korea) at gestational day 14 (G14) and housed individually in the animal facility. On G15, two doses of MAM (15 mg/kg intraperitoneally; MRIGlobal, Kansas City, MO, United states) were injected into pregnant rats, and normal saline was injected into controls at 830 and 1,830 h. Delivery occurred consistently on G21, which was considered postnatal day (P) 0 for the offspring. The overall experimental schedule is described in Figure 1.

### Measurement of Cortical Neurons, Morphological Analysis, and Immunoflorescence

The MAM-exposed rats and corresponding controls were transcardially perfused with 4% paraformaldehyde on P15 under deep anesthesia, and their brains were removed and cryoprotected. Serial coronal sections (20 μM thickness) were cut using a cryocut microtome and stored at −80°C for staining procedures.

For cresyl violet staining, the sections were fixed in 4% paraformaldehyde solution for 15 min and defatted in 70% ethanol solution containing 0.5% acetic acid for 5 min and washed. Thereafter, these sections were dipped in 0.3% cresyl violet solution for 3 min and washed.

A modified Golgi-Cox impregnation technique was performed using FD Rapid GolgiStain kit (FD NeuroTechnologies, Ellicott City, MD, United States) according to the manufacturer’s instructions. The brains of P15 rats were rinsed with distilled water to remove blood and trimmed to ∼1 cm thickness. The tissue was immersed in an impregnation solution for 2 weeks, transferred to solution C for 3 to 4 days, and cut into 120 μM-thick sections using a cryostat. The sections were mounted on silane-coated slides and stained with solutions D and E and dehydrated.

For immunofluorescence, six to eight sections were reacted per each rat and Supplementary Table S1 indicates the number of animals used in these experiments. The slides were washed and incubated in a blocking solution (containing 5% normal host serum and 0.1% BSA/0.3% Triton X-100) for 1 h after antigen retrieval (heating the slides on a hot plate at 50°C for 20 min). Primary antibodies [anti-MBP, a marker for myelinatin, 1: 500; anti-TBR1, for layer VI neurons; 1: 250, and anti-parvalbumin, for GABAergic neurons, 1: 500; Abcam, Cambridge, United Kingdom; anti-MAP2, for neuronal dendrites, 1: 1,000; anti-neuronal nuclei (NeuN), for neurons, 1: 500, and anti-reelin, for neuronal migration, 1: 1,000; EMD Millipore, MA, United States] with blocking solution were applied for 24 h at 4°C, and fluorescein-labeled secondary antibodies [fluorescein anti-mouse immunoglobulin G (IgG), fluorescein anti-rabbit IgG, and Cy^®^ 3 anti-rabbit IgG, Vector Laboratories; Alexa Fluor 594 anti-rat IgG, Thermo Fisher Scientific] were serially applied for 1 h at room temperature. DAPI (VECTASHIELD, Vector Laboratories, Inc., United States) counterstaining has performed.

All the images were acquired under a microscope (Olympus BX-53, Olympus corporation, Tokyo, Japan) with a digital camera (Olympus microscope digital camera, 5M CCD, Olympus corporation, Tokyo, Japan) and analyzed using Olympus cellSens standard 1.13 (Olympus corporation, Tokyo, Japan) and ImageJ software (NIH, Bethesda, MD, United States). The Golgi-impregnated staining was analyzed for a section per each animal at 100x and 200X magnification using Olympus CellSens standard 1.13 and ImageJ software. After taking picture for each focus at each magnification of the microscope, neurons were 3D-reconstructed by the Stack function of the ImageJ program. Stereological cell counting was done in selected area of the retrosplenial cortex (RSC) and the length of apical dendrites and the number of second branches of basal dendrites of the layer V pyramidal neurons were evaluated for two neurons per each animal. We analyzed apical dendrite length using Simple Neurite Tracer among Plugins of ImageJ, and basal dendrites were counted while looking directly with a microscope at a magnification of 400X. The pyramidal neurons were selected according to the following criteria: (1) triangular soma, (2) one apical dendrite, and (3) presence of basal dendrites; non-pyramidal neurons were counted as interneurons.

### Western Blot Analysis

For western blot analysis, bilateral cortical tissues from the bregma to posterior hippocampal area without the hippocampus (anteroposterior, 0 to −5 mM) were obtained from the control and MAM-exposed rats at P15. Isolated tissues from each rat were homogenized using PRO-PREP (iNtRON Biotechnology, Inc., Gyeonggi-do, Korea) at −4°C in an ice bath, and the protein samples were quantified using BSA. The acquired proteins were separated using SDS-PAGE and transferred to PVDF membranes. The membranes were blocked with 10% skim milk in tris-buffered saline with Tween-20 (TBST solution) for 1 h at room temperature. Thereafter, the membranes were incubated overnight at 4^°^C with the following primary antibodies: anti-NeuN (EMD Millipore, 1: 5,000), anti-GAD65 (EMD Millipore, 1: 5,000), and anti-parvalbumin (Abcam, 1: 2,000); anti-β-actin (Santa Cruz Biotechnology, Inc., Texas, United States, 1: 20,000) was used as a loading control (Supplementary Table S2). The membranes were then incubated in anti-rabbit IgG, HRP-linked antibody (Cell Signaling, Technology, Inc., MA, United States, 1: 10,000), or anti-mouse IgG, HRP antibody (Enzo Life Science, Inc., NY, United States, 1: 20,000) for 90 min at room temperature. The membranes were further rinsed with TBST and developed using an ECL solution (Clarity Western ECL substrate, Bio-Rad, CA, United States) on Fusion Solo S (Vilber Lourmat SAS, France). Normalization was performed by developing parallel western blots probed with β-actin antibody and analyzed via densitometry using Evolution-Capt (Fusion Software, Vilber Lourmat SAS).

### *In vivo* MRI Studies

Animals were maintained under anesthesia with 1% isoflurane in a 1:2 mixture of O_2_:N_2_O while monitoring their respiratory rate, electrocardiogram, and rectal temperature. MR images were acquired using a 7T/160 mM bore animal MRI system (PharmaScan, Bruker, Ettlingen, Germany) with the Paravision 6.0.1. software in a configuration comprising a 72 mM transmit volume coil and a mouse brain surface receiver coil.

Diffusion tensor imaging (DTI) was performed using a four-shot DT-echo planar imaging sequence (TR = 3.7s; TE = 20 ms; B0 = 1,000 s/mM^2^) with a 10 ms interval between the application of diffusion gradient pulses, a 4 ms diffusion gradient duration, a gradient amplitude of 46.52 mT/m, and Jones’ 30 gradient scheme. The fractional anisotropy (FA) maps of rats were calculated by using the Diffusion Toolkit software.^1^ For group comparison, we co-registered all FA images to the rat brain atlas using AFNI (the rat brain atlas in AFNI software, named mgh_wh_templete). The group FA-maps were obtained by averaging across subjects, and the group difference between MAM-exposed rats and control rats was calculated using unpaired, two-tailed *t*-test. The resulting difference t-maps were then thresholded at the FDR-corrected *p* < 0.01. All data analyses were processed using AFNI.

Glutamate chemical exchange saturation transfer (GluCEST) images were acquired and analyzed as previously reported in our group (Lee et al., 2018). GluCEST images were acquired using T_2_-weighted imaging (rapid acquisition with relaxation enhancement [RARE]) with a frequency selective saturation preparation pulse comprised a Gaussian pulse and a total duration of 1,000 ms (irradiation offset of 500.0 Hz and interpulse delay of 10 μs) at a B_1_ peak of 5.6 μT. Z-spectra were obtained from –5.0 ppm to +5.0 ppm with intervals of 0.33 ppm (total, 31 images). The sequence parameters were as follows: repetition time/echo time (TR/TE) = 4,200/36.4 ms, field of view = 30 × 30 mM^2^, slice thickness = 1 mM, matrix size = 96 × 96, RARE factor = 16, echo spacing = 6.066 ms, and average = 1. To measure the GluCEST (%), the regions of interest, manually drawn on the RSC in T_2_-weighted anatomical MR images, were overlaid on the GluCEST maps. GluCEST contrast is measured as the asymmetry between an image obtained with saturation at the resonant frequency of exchangeable amine protons (+3 ppm downfield from water for glutamate) and an image with saturation equidistant upfield from water (–3 ppm), according to the following equation:

$$\mathrm{GluCEST}\left(\%\right)=\frac{S_{-3.0\,p\,p\,m}-S_{+3.0\,p\,p\,m}}{S_{-3.0\,p\,p\,m}}*100$$

where *S*_–__3.0ppm_ and *S*_+3.0ppm_ are the magnetizations obtained with saturation at a specified offset from the water resonance of 4.7 ppm. The B0/B1 maps on the same slices were acquired for B_0_ and B_1_ correction. The B_0_ map was calculated by linearly fitting the accumulated phase per pixel following phase unwrapping against the echo time differences from gradient echo (GRE) images collected at TEs of = 1.9 and 2.6 ms. B_1_ maps were calculated by using the double-angle method (flip angles 30° and 60°) and the linear correction for B_1_ was calculated as the ratio of the actual B_1_ to the expected value.

**FIGURE 2:**
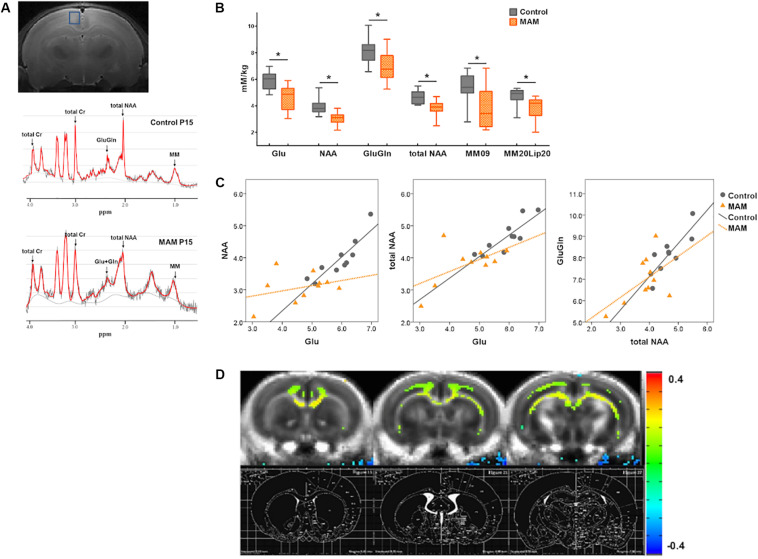
*In vivo* neuroimaging changes in rats with malformations of cortical development (MCDs) during infancy. **(A)** At postnatal day 15 (P15), regions of interest for MR spectroscopy data acquisition are depicted in the coronal planes in the retrosplenial cortex (RSC) (upper); an example of an MR spectrum is also shown (lower). **(B)** In infant rats with MCDs, glutamate (Glu), glutamate-plus-glutamine (GluGln), *N*-acetylaspartate (NAA), total NAA, macromolecule (MM), and lipid (Lip) levels are significantly lower (*n* = 10, Glu mean = 4.645, *SD* = 0.933, GluGln mean = 6.941, *SD* = 1.097, NAA mean = 3.069, *SD* = 0.472, total NAA mean = 3.819, *SD* = 0.61, MM09 mean = 3.829, *SD* = 1.561, MM20Lip20 mean = 3.82, *SD* = 0.9 *p* < 0.05) than those in controls (*n* = 10, Glu mean = 5.912, *SD* = 0.656, GluGln mean = 8.144, *SD* = 0.952, NAA mean = 3.952, *SD* = 0.625, total NAA mean = 4.654, *SD* = 0.516, MM09 mean = 5.364, *SD* = 1.118, MM20Lip20 mean = 4.71, *SD* = 0.652). **(C)** A significant positive correlation is observed between Glu and NAA, Glu and total NAA, and total NAA and GluGln when the partial correlation analysis is performed by controlling group differences (Glu and NAA levels: *r* = 0.563, *p* = 0.012, *df* = 17; Glu and tNAA levels: *r* = 0.676, *p* = 0.001, *df* = 17; total NAA and GluGln levels: *r* = 0.664, *p* = 0.002, *df* = 17). When the correlation analysis was done in each group, controls showed significant positive correlation of glutamate and NAA levels (*r* = 0.903, *p* = 0.000), glutamate and total NAA levels (*r* = 0.855, *p* = 0.002), and total NAA and GluGln levels (*r* = 0.794, *p* = 0.006) but there is no significant correlation of these neurometabolites in MCD rats (glutamate and NAA levels: *r* = 0.248; *p* = 0.489; glutamate and total NAA levels: *r* = 0.442; *p* = 0.200; total NAA and GluGln levels: *r* = 0.418; *p* = 0.229). **(D)** Subtraction FA-maps (control rats minus MAM-exposed rats) are shown over control-group-averaged FA-map (threshold FDR-corrected *p* < 0.01). The rat brain atlas defined by Paxinos and Watson (2014) was also shown for the references. The MAM-exposed rats showed significant reduction of FA values in the cingulate cortex, corpus callosum, cingulum, and deep white matter including external capsule (control; *n* = 16, MAM; *n* = 15). *Statistically significant, *p* < 0.05.

^1^H-MRS was performed at P15 in MAM-exposed rat (*n* = 10) and control (*n* = 10). The MR spectra were acquired through a signal voxel (from bregma to –3.0 mM in a coronal section, 1.2 × 1.3 × 3 mM^3^; Figure 2A) in the RSC using a point-resolved spectroscopy (PRESS) sequence for 128 acquisitions with TR/TE = 5,000/13.4 ms. For quantification, unsuppressed water signals were also acquired from the same voxel (average = 8). The water-suppressed time domain data were analyzed between 0.2 and 4.0 ppm, without further T1 or T2 correction. For quantification, all the MR spectra were processed with the linear combination analysis method (LC Model ver. 6.0, Los Angeles, CA, United States) and absolute metabolite concentrations (mmol/kg wet weight) were calculated using the unsuppressed water signal as an internal reference (assuming 80% brain water content; Terpstra et al., 2010; Lee et al., 2018). The *in vivo* proton spectra were judged to have an acceptable value if the standard deviation of the fit for the metabolite was less than 20%.

### Statistical Analysis

Statistical analyses were performed using IBM SPSS Statistics for Windows, Version 22.0 (IBM Corp., Armonk, NY, United States). The level of significance was preset to *p* < 0.05. Two-group comparisons of the concentrations of metabolites and cortical neuron analysis were performed using the Mann-Whitney *U*-test. Student’s *t*-test was used for protein expression data following normal distribution in two-group comparisons. Spearman’s correlation analysis was used to analyze the correlation between GluCEST (%) and cortical length or several neurometabolites. To control the group differences, partial correlation analysis was used to determine the associations among neurometabolites in control and MAM-exposed rats. We planned studies of a continuous response variable from independent control and experimental subjects with 1 control(s) per experimental subject. In previous experiments, the responses within each subject group was normally distributed for these experiments. Using preliminary data means with standard deviations, we extracted the number of animals we needed to be able to reject the null hypothesis that the population means of the experimental and control groups are equal with probability (power) 0.95. The Type I error probability associated with this test of this null hypothesis is 0.05. The number of animals in each group were 4 or more for MRS and GluCEST imaging, 9 for Golgi staining, 14 for WB and IF staining.

## Results

### Cortical Neuronal Paucity and Dendritic Arborization Failure in the RSC of the Infant Rat Model of MCDs

**FIGURE 3:**
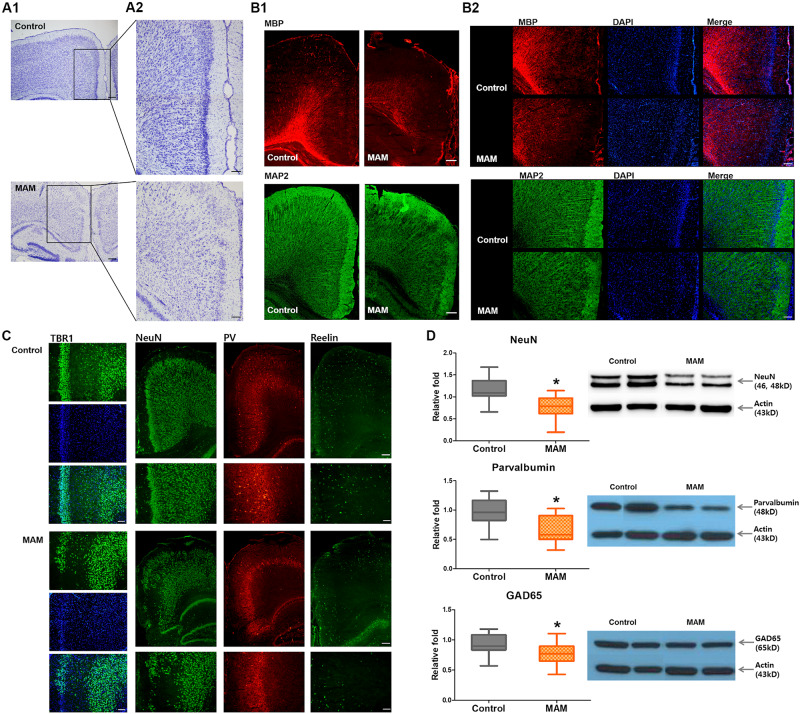
Microstructural changes in the retrosplenial cortex (RSC) and deficiency of cortical interneurons in infant rats with malformations of cortical development (MCDs). **(A)** Pathological alteration of the cortical structure is shown by cresyl violet staining. Scale bars: **(A1)** 200 μM; **(A2)** 100 μM. **(B)** Immunofluorescence studies show sparser MBP staining and collapsed microstructures with MAP2. Scale bars: **(B1)** 200 μM; **(B2)** 100 μM. **(C)** In infant rats with MCDs, TBR1, neuronal nuclei (NeuN), parvalbumin (PV), and reelin expressions in the RSC are scantier than those in controls. Scale bars: lower, 200 μM; upper, 100 μM. **(D)** Western blot analysis shows significant reductions of NeuN (control; *n* = 32, mean = 1.174, *SD* = 0.232, MAM; *n* = 24, mean = 0.769, *SD* = 0.247, *p* = 0.000), PV (control; *n* = 23, mean = 0.979, *SD* = 0.231, MAM; *n* = 19, mean = 0.65, *SD* = 0.217, *p* = 0.000), and GAD65 (control; *n* = 30, mean = 0.928, *SD* = 0.171, MAM; *n* = 22, mean = 0.77, *SD* = 0.186, *p* = 0.003) in the cortex of rats with MCDs. MAM, methylazoxymethanol. *Statistically significant, *p* < 0.05.

Cresyl violet staining showed collapsed cortical structures in infant rats prenatally exposed to MAM (Figure 3A). Immunofluorescence staining of the RSC were qualitatively evaluated and MBP, MAP2 immunoreactivity and NeuN-, parvalbumin-, GAD65- positive neuronal populations were sparser in MAM-exposed infant rats than in controls (Figures 3B,C). There were relatively scantier reelin immunoreactivity in RSC of MAM-exposed animals and TBR1 (+) neurons dispersed more widely and the layer VI was obliterated in MAM-exposed rats compared to those in controls (Figure 3C). The protein expressions of NeuN, parvalbumin, and GAD65 were also significantly decreased in rats with MCDs (NeuN, *p* = 0.000; parvalbumin, *p* = 0.000; GAD65, *p* = 0.003; Figure 3D).

**FIGURE 4:**
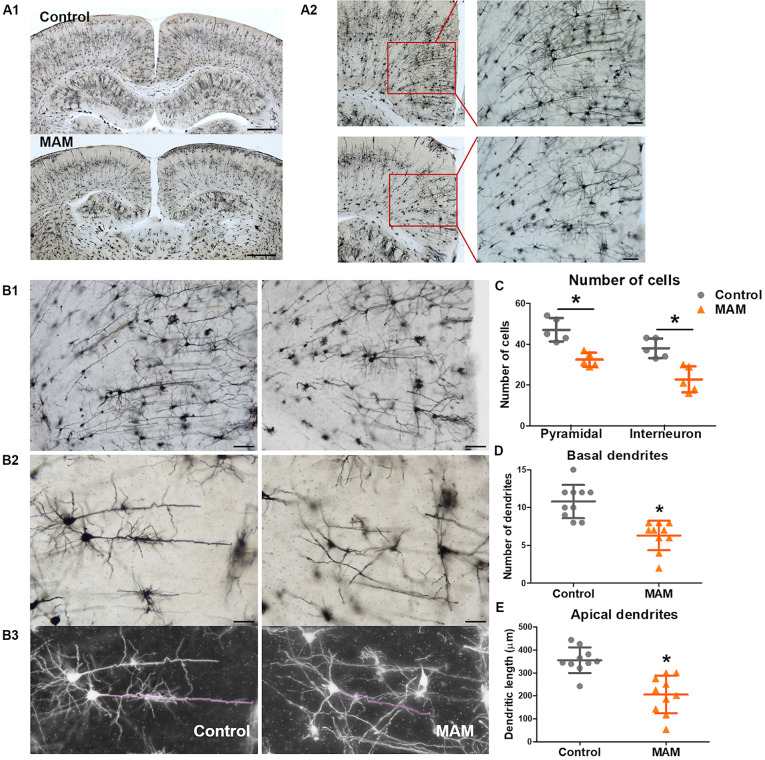
Dendritic arborization of retrosplenial cortex (RSC) pyramidal cells in an infant rat model of malformations of cortical development (MCDs). **(A)** Golgi staining reveals cortical neuronal deficits in infant rats with MCDs (control; *n* = 5, MAM; *n* = 5). **(B,C)** In the RSC, the numbers of pyramidal cells and interneurons are significantly reduced (control; *n* = 5, mean = 47.0, *SD* = 5.701, mean = 38.0, *SD* = 4.796, MAM; *n* = 5, mean = 32.4, *SD* = 3.435, mean = 22.8, *SD* = 6.380, *p* = 0.009). **(D,E)** The number of basal dendrites of pyramidal cells and the length of apical dendrites are significantly reduced (control; *n* = 10, mean = 10.8, *SD* = 2.201, mean = 356.066, *SD* = 55.919, MAM; *n* = 10, mean = 6.3, *SD* = 1.947, mean = 206.131, *SD* = 81.888). Scale bars: **(A1)** 1 mM; **(A2)** 100 μM; **(B1)** 100 μM; **(B2)** 50 μM. MAM, methylazoxymethanol. *Statistically significant, *p* < 0.05.

Golgi staining also showed a significantly smaller number of cortical neurons in infant rats with MCDs (*n* = 5) than in controls (*n* = 5, *p* = 0.009; Figure 4C). The pyramidal neurons of rats with MCDs exhibited shorter apical dendrites as well as a smaller number of basal dendrites than did controls (*n* = 10; apical dendrites, *p* = 0.001; basal dendrites, *p* = 0.000; Figures 4D,E).

### *In vivo* MR Structural and GluCEST Imaging in Rats With MCDs During Infancy

**FIGURE 5:**
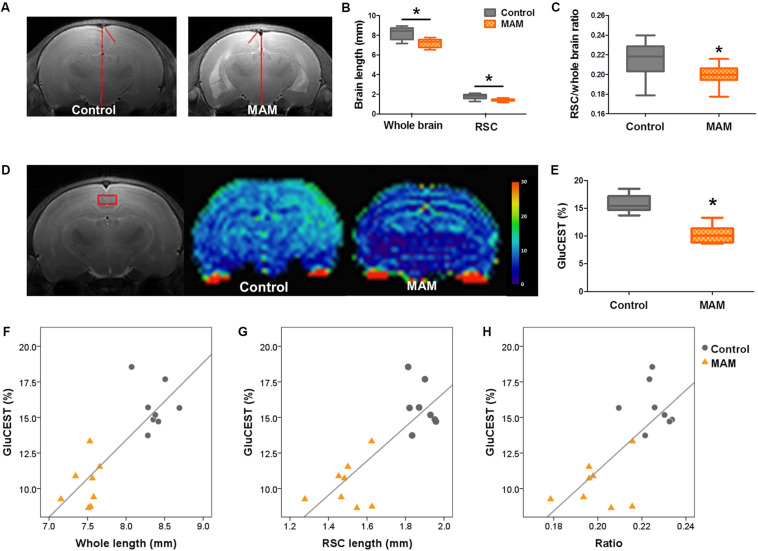
*In vivo* MR structural and GluCEST imaging in rats with MCDs during infancy. **(A)** In representative T2-weighted MR images, the length of the whole brain and retrosplenial cortex (RSC) are measured (red lines) at 3 mM posterior to the bregma. **(B)** In infant rats with malformations of cortical development (MCDs), the length of the whole brain and RSC, and **(C)** the ratio of the RSC to cortex are significantly decreased (methylazoxymethanol [MAM], *n* = 23, mean = 7.194, *SD* = 0.435, mean = 1.433, *SD* = 0.132, mean = 0.199, *SD* = 0.011; Control, *n* = 24, mean = 8.215, *SD* = 0.562, mean = 1.783, *SD* = 0.235, mean = 0.216, *SD* = 0.016; *p* = 0.000). **(D,E)** Glutamate chemical exchange saturation transfer (GluCEST) level (%) measured from the RSC (red box) is more significantly reduced in rats with MCDs (*n* = 8, mean = 10.309, *SD* = 1.616) than in controls (*n* = 8, mean = 15.761, *SD* = 1.599, *p* = 0.000). **(F–H)** A significant positive correlation is observed between GluCEST (%) and cortical length when the two groups are combined (**F:** *r* = 0.785, *p* = 0.000; **G:** *r* = 0.685, *p* = 0.003; **H:** *r* = 0.688, *p* = 0.003). *Statistically significant, *p* < 0.05.

*In vivo* MRI at P15 also showed the anatomical changes caused by prenatal MAM exposure, which were comparable to the pathological changes. The dorsal-to-ventral whole-brain length and RSC length were significantly lesser in infant rats with MCDs (*n* = 23, *p* = 0.000; Figures 5A–C) than in controls (*n* = 24).

The RSC of MAM-exposed rats (*n* = 8) showed significantly lower GluCEST (%) than that of controls at P15 (*n* = 8, *p* = 0.000; Figures 5D,E and Supplementary Figure S1). A significant positive correlation was also observed between whole-brain length, cortical length, cortical brain length ratio, and GluCEST (%) when the rats prenatally exposed to MAM and controls were combined (Figure 5F: *r* = 0.785, *p* = 0.000; **G**: *r* = 0.685, *p* = 0.003; **H**: *r* = 0.688, *p* = 0.003).

### *In vivo* Neurometabolic/DTI Changes in the RSC of the Infant Rat Model of MCDs

Infant rats with MCDs showed microstructural and neurometabolic changes corresponding to the pathological data. MRS analysis focusing on the RSC showed a significant reduction in neurometabolite levels, including those of glutamate (*p* = 0.002), glutamate-plus-glutamine (*p* = 0.017), NAA (*p* = 0.002), total NAA (*p* = 0.004), macromolecule 09 (*p* = 0.024), and macromolecule 20-plus-lipid 20 (*p* = 0.027), in infant rats with MCDs (*n* = 10) than in controls (*n* = 10; Figure 2B). A significant positive correlation was also observed between glutamate and NAA, glutamate and total NAA, as well as total NAA and glutamate-plus-glutamine when the partial correlation analysis was performed by controlling group differences (glutamate and NAA levels: *r* = 0.563, *p* = 0.012, *df* = 17; glutamate and total NAA levels: *r* = 0.676, *p* = 0.001, *df* = 17; total NAA and glutamate-plus-glutamine levels: *r* = 0.664, *p* = 0.002, *df* = 17, Figure 2C). When the correlation analysis was done in each group, control group showed significant positive correlation of these glutamate and NAA (*r* = 0.903, *p* = 0.000), glutamate and total NAA (*r* = 0.855, *p* = 0.002), and total NAA and glutamate-plus-glutamine levels (*r* = 0.794, *p* = 0.006) but MCD rats did not show correlation of these metabolites (glutamate and NAA: *r* = 0.248, *p* = 0.489; glutamate and total NAA: *r* = 0.442, *p* = 0.200; total NAA and glutamate-plus-glutamine: *r* = 0.418, *p* = 0.229).

Microstructural analysis also revealed decreased FA values in the cingulate cortex, corpus callosum, cingulum, and deep white matter including external capsule in MAM-exposed rats at P15 (MAM-exposed rats, *n* = 15; controls, *n* = 16; Figure 2D).

## Discussion

MCDs, which show the disrupted developmental process of the brain, are closely related to intractable epilepsy and developmental delay (Barkovich et al., 2012; Kuzniecky, 2015; Becker and Beck, 2018). Many studies have shown that MAM-induced MCD rats have structural abnormalities similar to those observed in patients with MCDs, but most studies have focused on adult rats and pathologic changes (Moore et al., 2006; Lodge and Grace, 2009; Chin et al., 2011). As epilepsy associated with MCDs often occurs during infancy and is frequently refractory to current treatments (Tassi et al., 2002; Kang et al., 2013), we tried to explore the early developmental changes of MCDs during infancy.

Although neocortical structures in the early developmental period have not been extensively investigated in this rat model, the altered firing properties of their neuronal subpopulations at 3–7 weeks of age and abnormal neocortical electrical activities at P15 have been reported (Chevassus-Au-Louis et al., 1998; Kim et al., 2017). To demonstrate the abnormal neocortical neuronal migration at early stages, we selected the RSC for pathological investigation in this study. In imaging studies, including MRS, on small animals using regions of interest, the RSC is relatively more easily recognizable and accessible than are other neocortical structures. The RSC is known to be associated with the default mode network and cognitive functions such as navigation, learning, and memory (Sugar et al., 2011).

In young rats prenatally exposed to MAM, we could show the histopathologic features of MCDs (Garbossa and Vercelli, 2003; Pang et al., 2008; Barkovich et al., 2012; Kuzniecky, 2015), such as the collapse of the cortex layer, alteration of cortical structures, hypomyelination, and abnormalities of microtubule formation (Figure 3). Golgi staining of the RSC also showed reduced numbers of cortical neurons and dendritic arborization of pyramidal cells in infant rats with MCDs (Figure 4). This result is consistent with that of a previous study on MAM-induced pathological changes in the brains of Wistar albino rats at P12 and adulthood (Garbossa and Vercelli, 2003). In addition, a significant reduction in parvalbumin-positive cells was identified in the RSC (Figures 3C,D). Although inhibitory interneurons account for a relatively small proportion of cortical cells, inhibitory cortical neuronal dysfunction is known to cause various neurological diseases, including epilepsy and autism, since these neurons play an important role in the cortical network (Kelsom and Lu, 2013; Wamsley and Fishell, 2017). Previous studies have reported that other MAM-induced MCD models showed distorted functional connection of hippocampal-neocortical neurons (Chevassus-Au-Louis et al., 1998) and alteration of interneuron migration due to aberrant GABA_A_ activity in the neocortex (Abbah and Juliano, 2014). In this study, prenatally MAM-exposed rats also showed decreased GAD65 expression in the RSC, which may be consistent with altered GABAergic activity in the neocortex (Ji et al., 1999; Stork et al., 2000; Silva et al., 2002; Wieronska et al., 2010). Furthermore, abnormal TBR1 immunoreactivity and significant reduction of reelin, which regulates neuronal migration processes by controlling cell-cell interactions (Niu et al., 2008; Wieronska et al., 2010; Lee and D’Arcangelo, 2016; Wasser and Herz, 2017), were observed in this model at P15. These overall failures of cortical migration and dendritic arborization of pyramidal cells, as well as insufficient numbers of inhibitory interneurons, may result in the seizure susceptibility of this model during infancy. However, no dysmorphic neurons or balloon cells are observed in these young rats with MCDs, and these pathologic findings are consistent with the International League Against Epilepsy classification of FCD type I (Blumcke et al., 2011), which is rarely delineated using current imaging techniques.

On the basis of these pathological findings, we also tested whether the MCDs in infancy can be diagnosed through *in vivo* imaging techniques in clinical settings. We observed a quantifiable reduction in RSC length and whole-brain length, as previously shown in this model (Kim et al., 2017), which are potential biomarkers of FCD in clinical settings (Figures 5A–C). A new imaging technique involving glutamate measurement, GluCEST imaging, also revealed significantly decreased glutamate in the RSC, and 1H-MRS analysis supported this finding by showing a significant reduction in glutamate and glutamate-plus-glutamine in the RSC (Figures 2B, 5D,E). A significant correlation was also observed between GluCEST (%) and RSC length/whole-brain length, suggesting GluCEST (%) was a potential biomarker of FCD (Figures 5F–H). GluCEST imaging is emerging molecular MRI technique with higher spatial resolution than MRS, potentially allowing for more precise visualization of the excitatory network of high glutamate concentrations (Davis et al., 2015). In addition to its crucial function in cognition (Pepin et al., 2016), glutamate is a major excitatory neurotransmitter in the central nervous system that is closely associated with epilepsy (Eid et al., 2016) and glutamate also has key roles in the radial migration of pyramidal neurons as well as tangential migration (Luhmann et al., 2015). Thus, this neurometabolic profile found in the present study can be indirectly associated with the abnormal cortical migration observed in the MAM-induced MCD rats at P15 and can be a useful marker of epileptic foci (Davis et al., 2015). However, there are several confounding factors in interpreting GluCEST results including the signal contributions from amines in proteins and nuclear Overhouser effects (NOE) (Cui and Zu, 2020). Thus, the changes in GluCEST in these rats with MCD can be caused by the changes in content or conformation of proteins in these pathological tissues as well as glutamate. Abnormal neuronal development in malformed cortex as well as the dendritic arborization failures in this study (Andreae and Burrone, 2015) can be associated with these *in vivo* imaging changes of GluCEST and decrease of cortical thickness.

Moreover, *in vivo* ^1^H-MRS also showed findings comparable with the pathological changes of the MCD cortex. Reduced NAA, macromolecules and lipid levels were observed in the RSC of rats prenatally exposed to MAM (Figure 2B), which was consistent with the pathological reduction in cortical neurons as well as decreased dendritic arborizations identified in this study (Figures 3, 4) and previous human studies (Woermann et al., 2001; Mueller et al., 2005; Blüml and Panigrahy, 2013). Additional analysis showed a clear positive correlation between glutamate and NAA, glutamate and total NAA, as well as total NAA and glutamate-plus-glutamine in the RSC of control rats at P15. However, these correlations were obscured in rats prenatally exposed to MAM, thus suggesting the disrupted neuronal development of the RSC in these rats.

A significant reduction in FA was observed in the cingulate cortex, corpus callosum, cingulum, and deep white matter including external capsule of the rats prenatally exposed to MAM in this study; this finding is also observed in patients with FCD (Lee et al., 2004; Donkels et al., 2017). The measure of anisotropy reflects changes in myelination, dendritic architecture of the cortical neuron, and fiber connection (Huppi and Dubois, 2006). FA is considered to be an indirect indicator of myelination and is generally increases with advancing age (Semple et al., 2013). In this early developmental period P15, the corpus callosum, cingulum, external capsule is in rapid myelinating process (Downes and Mullins, 2014) and impaired developmental process in MAM-exposed rats may attribute the significant reduction of FA in these areas. Therefore, FA reduction in the RSC, cingulum and corpus callosum of this MCD model, reflecting the malformed brain with white matter paucity and abnormal dendritic arborization, can be a biomarker of white matter deterioration. Overall, these unconventional *in vivo* imaging data obtained in this study could reflect the pathological changes in MCDs and can be used in clinics to improve the diagnosis of focal cortical abnormalities in patients with intractable epilepsy.

This study showed deficits of cortical interneurons and dendritic arborization failures in the cortex of rats prenatally exposed to MAM at P15 and corresponding *in vivo* MRI characteristics by using new imaging techniques. These MRI characteristics should be further validated as diagnostic biomarkers of MCD.

## Data Availability Statement

All datasets generated for this study are included in the article/Supplementary Material.

## Ethics Statement

The animal study was reviewed and approved by the Institutional Animal Care and Use Committee of the Ulsan University College of Medicine and conformed to the Revised Guide for the Care and Use of Laboratory Animals (8th Edition, 2011).

## Author Contributions

ML and M-SY contributed to the conception, design of the study, and drafting the manuscript and figures. ML and E-JK contributed to the acquisition of data. ML, E-JK, D-CW, W-HS, and M-SY contributed to the analysis of data.

## Conflict of Interest

The authors declare that the research was conducted in the absence of any commercial or financial relationships that could be construed as a potential conflict of interest.

## Footnotes

1 http://trackvis.org/
