# Supplementary material for: In vivo MRI Successfully Reveals the Malformation of Cortical Development in Infant Rats
Source: Front Neurosci. 2020 May 20;14:510. doi: 10.3389/fnins.2020.00510 (PMC7251149; doi:10.3389/fnins.2020.00510)
Supplement: Supplementary file 1 [file Image_1.pdf]

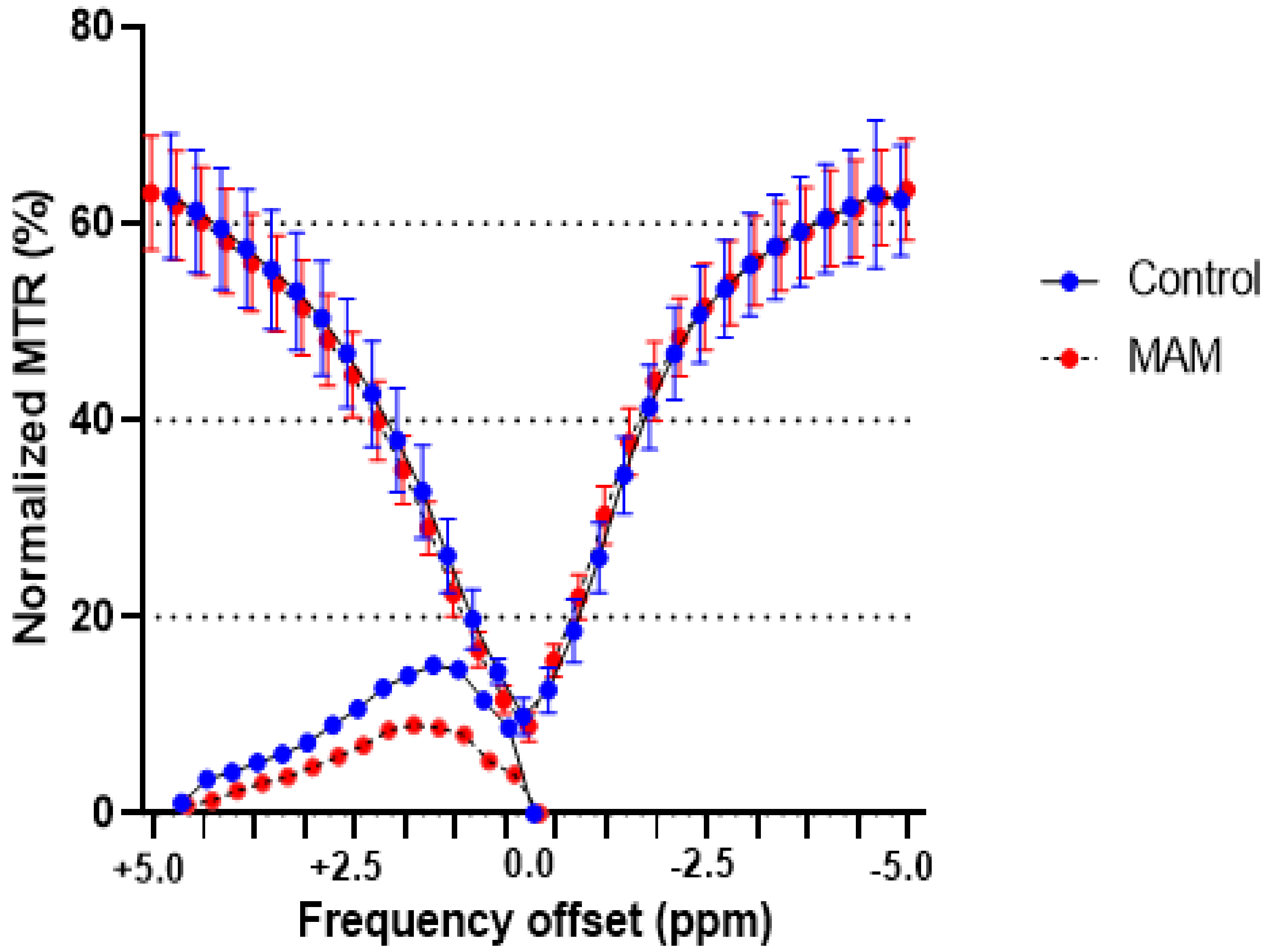

Supplementary figure 1. The experimentally acquired Z-spectral (Z+1400 to –1400 Hz) signal intensity from retrosplenial cortex of MAM-exposed rats and controls are shown. MTRasym (%) curves of each rats are shown at the bottom left.

MTRasym = [Msat(-Δω) − Msat(+Δω)]/M0.

"Msat (± Δω) are the magnetizations obtained with saturation Δω offset from the water resonance and M0 is the magnetization without saturation."
