# Supplementary material for: In vivo MRI Successfully Reveals the Malformation of Cortical Development in Infant Rats
Source: Front Neurosci. 2020 May 20;14:510. doi: 10.3389/fnins.2020.00510 (PMC7251149; doi:10.3389/fnins.2020.00510)
Supplement: Supplementary file 2 [file Table_1.pdf]

Supplementary Table 1 (Table S1). The number of litters used in each experiment.

In vivo techniques

	1H-MRI/MRS		DTI		GluCEST		Cortical length	
	Control	MAM	Control	MAM	Control	MAM	Control	MAM
Number of Litters	2	3	3	5	1	1	4	6
Number of animals	10	10	16	15	8	8	24	23

In vitro experiments

	Western blot		Immunofluorescence & Cresyl violet		Golgi stain	
	Control	MAM	Control	MAM	Control	MAM
Number of Litters	4	5	4	4	2	2
Number of animals	32	24	14	11	5	5

Litter: a group of pups born from one mother

Supplementary Table 2 (Table S2). The list of antibodies used.

Western blot

Antibody	Host#	Clonality*	Catalog No.	Company
GAD65	Rb	Unknown	ABN101	Millipore
NenN	M	M	MAB377	Millipore
Parvalbumin	Rb	P	ab11427	Abcam
β-Actin	M	M	sc-47778	Santa Cruz

#Host: M, mouse; Rb, rabbit

*Clonality: M, monoclonal antibody; P, polyclonal antibody

Immunofluorescence

Antibody	Host#	Clonality*	Catalog No.	Company
Myelin Basic Protein Ab	Rat	M	ab7349	Abcam
MAP-2	Rb	P	AB5622-1	Millipore
NenN	M	M	MAB377	Millipore
Parvalbumin	Rb	P	ab11427	Abcam
Reelin	M	M	MAB5364	Millipore
TBR1	R	M	AB183032	Abcam

#Host: M, mouse; Rb, rabbit

*Clonality: M, monoclonal antibody; P, polyclonal antibody
